# Ulnar styloid abduction angle: a novel radiological predictor of Palmer Type IB tears of the triangular fibrocartilage complex

**DOI:** 10.3389/fmed.2026.1842984

**Published:** 2026-07-03

**Authors:** Wanxue Wang, Yixin Zhang, Junmiao Liu, Heming Xu, Xiaoyang Xu, Peng Chen, Haipeng Liu, Haitao Fu, Xia Zhao, Chao Qi

**Affiliations:** 1 Department of Sports Medicine, The Affiliated Hospital of Qingdao University, Qingdao, Shandong, China; 2 Department of Epidemiology and Health Statistics, Public Health College, Qingdao University, Qingdao, Shandong, China

**Keywords:** anatomy, imaging, predictors, radiocarpal joint, TFCC tear, ulnar styloid process

## Abstract

**Background:**

Deep ulnar fossa tears of the triangular fibrocartilage complex (TFCC) frequently lead to instability of the radioulnar joint. Although positive Ulnar Variance (UV) is a recognised longitudinal bony factor associated with TFCC injury, many tears still occur in patients with neutral or negative UV; the influence of the morphological orientation of the ulnar styloid process in the coronal plane on ligament susceptibility remains unclear.

**Objective:**

To assess whether the ulnar styloid abduction angle (USAA) is an independent bony predictor of Palmer Type IB TFCC tears, and to investigate its non-linear dose–response relationship and clinical predictive value.

**Study design:**

Retrospective case–control study; evidence grade: Level 3.

**Methods:**

This study included 362 patients who met strict radiographic quality control criteria in true posteroanterior (PA) views, comprising 110 cases of Palmer Type IB tears confirmed by arthroscopy and 252 control cases with intact TFCC confirmed by 3.0 T MRI and clinical follow-up. Missing values were handled using the Multiple Imputation by Chained Equations (MICE) method, and 1:1 propensity score matching (PSM) was performed to balance baseline characteristics. Multivariate binary logistic regression was applied to identify independent predictors. A restricted cubic spline (RCS) model was used to assess non-linear associations, and the predictive robustness and clinical net benefit of the model were evaluated via Bootstrap resampling for internal validation and decision curve analysis (DCA).

**Results:**

The USAA was significantly greater in the case group than in the control group (21.0° ± 4.0° vs. 14.8° ± 3.8°, *p* < 0.001). After full adjustment for propensity score matching (PSM) and multivariate factors, increased USAA remained independently associated with Palmer Type IB tears (adjusted odds ratio [aOR] = 1.37 per degree; 95% CI, 1.25 to 1.51; *p* < 0.001) and was independent of the effect of ulnar variance. RCS analysis revealed a significant non-linear threshold effect (overall non-linearity *p* = 0.012); when USAA exceeded 17.5°, the risk of rupture showed a steep exponential increase. The area under the receiver operating characteristic curve (AUC) after optimism-corrected AUC using bootstrap resampling was 0.849. The optimal cut-off value of ≥17.5° yielded a sensitivity of 81.8% and a specificity of 78.2% for identifying tears. DCA confirmed that the USAA-based diagnostic strategy offers significant net clinical decision-making benefits across a wide range of threshold probabilities.

**Conclusion:**

An increased USAA is an independent and highly significant two-dimensional radiographic predictor of Palmer Type IB TFCC tears, exhibiting a non-linear dose–response relationship with the risk of injury. This intrinsic morphological variation in the coronal plane may reshape the biomechanical tolerance threshold of the deep fibres, with a pathogenic mechanism independent of classic axial ulnar variance.

**Clinical implications:**

USAA can be measured cost-effectively and reliably on standard wrist radiographs. A USAA ≥ 17.5° may serve as an objective screening radiographic parameter for first-line clinical screening, aiding in the optimisation of early risk stratification and clinical triage pathways for patients with wrist ulnar pain.

## 1 Introduction

Injuries to the triangular fibrocartilage complex (TFCC) are one of the most common causes of ulnar-sided wrist pain and dysfunction (1, 2). As the primary intrinsic stabilising structure of the distal radioulnar joint (DRUJ) and the weight-bearing cushion of the radiocarpal joint, the anatomical integrity of the TFCC is crucial for normal wrist kinematics (3, 4). In particular, the deep proximal component of the TFCC, namely the ulnar-radial ligament, is firmly attached to the fovea at the base of the ulnar styloid process (5). The widely adopted Palmer classification categorizes TFCC lesions into two primary groups: traumatic (Type I) and degenerative (Type II) tears. Traumatic Type I tears are further subdivided based on the specific anatomical site of the rupture (A to D). Within this framework, injuries involving avulsion at this fovea attachment site are defined as Palmer Type IB tears (6). Injury to this anatomical region, namely, a Palmer Type IB tear, results in significant kinematic trajectory abnormalities of the DRUJ during pronation and supination of the wrist. Unlike peripheral simple tears, which cause only localised pain, rupture of the deep fibres of the fovea ulnaris leads to significant biomechanical instability of the DRUJ during pronation and supination, thereby severely affecting the patient’s grip strength and forearm rotation function, and typically requiring arthroscopic repair surgery (7, 8). Therefore, identifying potential anatomical factors that are associated with this specific type of tear is of great significance for early intervention in high-risk populations and the formulation of clinical management strategies.

In exploring bony structural predisposing factors for TFCC injuries, previous studies have primarily focused on longitudinal changes in ulnar length. The UV variant, due to its significant increase in axial load transmission at the radiocarpal joint, has been widely recognised as a classic bony predictor for ulnar impaction syndrome and secondary degenerative TFCC tears (9). However, clinical observations indicate that a significant proportion of traumatic or microtraumatic Palmer Type IB tears occur in patients with neutral or even negative ulnar variance (10, 11). This clinical phenomenon suggests that the UV alone, as a single longitudinal parameter, is insufficient to fully explain the structural vulnerability of the TFCC’s ulnar fossa attachment site; other, as yet insufficiently recognised, coronal or sagittal anatomical variations must necessarily play a role.

The ulnar styloid process is not only an important radiological marker but also a robust bony anchor connecting the deep and superficial fibres of the TFCC (12, 13). Although a substantial body of literature has confirmed a clear association between fractures of the base of the ulnar styloid process and acute DRUJ instability (14, 15), there has been little research into the influence of the morphology and orientation of an anatomically intact natural ulnar styloid process on the biomechanical environment of the TFCC. We propose a novel anatomical hypothesis: the inherent angulation of the ulnar styloid process relative to the shaft of the ulna in the coronal plane may directly influence the tensile force vector at the ulnar fovea. During high-load composite movements involving extreme wrist rotation combined with ulnar deviation, such as those seen in racket sports (16), excessive radial abduction of the ulnar styloid process may alter the isometric alignment of the deep ligamentous fibres. This structural alteration may cause the insertion site to bear abnormal shear stress rather than the tensile stress observed under physiological conditions (17), thereby making individuals more susceptible to Palmer Type IB tears when subjected to subthreshold loads.

To date, there are no standardised radiographic parameters for quantifying the natural orientation of the ulnar styloid process in the coronal plane, and its clinical relevance to TFCC injuries remains unknown. Therefore, the aim of this study was to define a new radiographic parameter, the ulnar styloid abduction angle (USAA), on standard posterior–anterior (PA) wrist radiographs and to investigate its diagnostic value through a large-scale cohort study. We hypothesize that an increased USAA will significantly increase susceptibility to deep TFCC tears and that this parameter is an independent bony indicator for predicting Palmer Type IB tears, with its predictive performance unaffected by traditional ulnar variance.

## 2 Methods

### 2.1 Study design and ethical review

This study is a single-centre retrospective case–control study. Prior to the commencement of the study, the study protocol was formally approved by the institution’s Ethics Review Committee (QYFYWZLL42069). Given the retrospective nature of the study, the committee waived the requirement for informed consent from patients. Through a search of the hospital’s electronic medical records and imaging databases, adult patients who presented with ulnar wrist pain between January 2020 and December 2025 and underwent high-resolution magnetic resonance imaging (MRI) or arthroscopy of the wrist were preliminarily screened.

### 2.2 Patient selection and grouping criteria

To minimise validation bias arising from diagnostic variability, this study established strict homogenisation, inclusion, and exclusion criteria. The inclusion criteria for the case group were as follows: (1) Age between 18 and 65 years; (2) A definitive diagnosis of a simple deep fibre avulsion tear of the ulnar notch of the triangular fibrocartilage complex (TFCC) (Palmer Type IB tear), confirmed by wrist arthroscopy; (3) Availability of preoperative anteroposterior (PA) wrist X-rays meeting quality control standards.

Inclusion criteria for the control group (TFCC-intact group) included: (1) Aged between 18 and 65 years; (2) Confirmed as having an intact TFCC structure following independent review by at least two musculoskeletal radiologists with over 10 years’ experience. To reduce the false-negative rate, MRI diagnosis in the control group must be based on images obtained using a 3.0 T high-resolution MRI scanner in conjunction with a dedicated wrist surface coil; (3) Possession of a contemporaneous standard PA wrist X-ray; (4) Electronic medical record follow-up data indicating that, for at least 6 months following the MRI examination, the patient had not undergone any targeted injections or specialist surgical interventions due to exacerbation of ulnar-wrist pain, thereby providing clinical evidence to confirm the structural stability of the deep ligaments.

Exclusion criteria: candidates in both groups were excluded if any of the following conditions were present: (1) Arthroscopy or MRI confirming a central perforation of the TFCC (Palmer Type IA), a palmar or ulnar-carpal ligament tear (Palmer Type IC), a radial avulsion (Palmer Type ID) or a degenerative tear (Palmer Type II); (2) A history of forearm fractures, such as those of the distal radius or ulnar shaft; (3) Imaging-confirmed osteoarthritis of the radioulnar joint, cystic changes associated with ulnar impingement syndrome, or Madelung deformity; (4) A history of previous wrist surgery.

### 2.3 Imaging assessment and ‘true posterior–anterior’ quality control

All imaging parameters were measured using the hospital’s Picture Archiving and Communication System (PACS). Two specialist-trained orthopaedic surgeons independently performed all X-ray measurements without knowledge of the patients’ clinical groupings. To mitigate projection distortion caused by forearm rotation when assessing three-dimensional structures on two-dimensional X-ray images, this study established strict radiographic quality control criteria for the “True PA” view. Images were included in the analysis only if they met the following criteria: (1) The projection of the extensor carpi ulnaris (ECU) tendon groove must be situated at the standard anatomical neutral centre of the base of the ulnar styloid; (2) The radial-ulnar overlap at the distal radioulnar joint space must be normal (<2 mm). Any radiograph exhibiting an eccentric ulnar styloid process contour due to latent forearm pronation or supination was systematically excluded.

**Figure 1:**
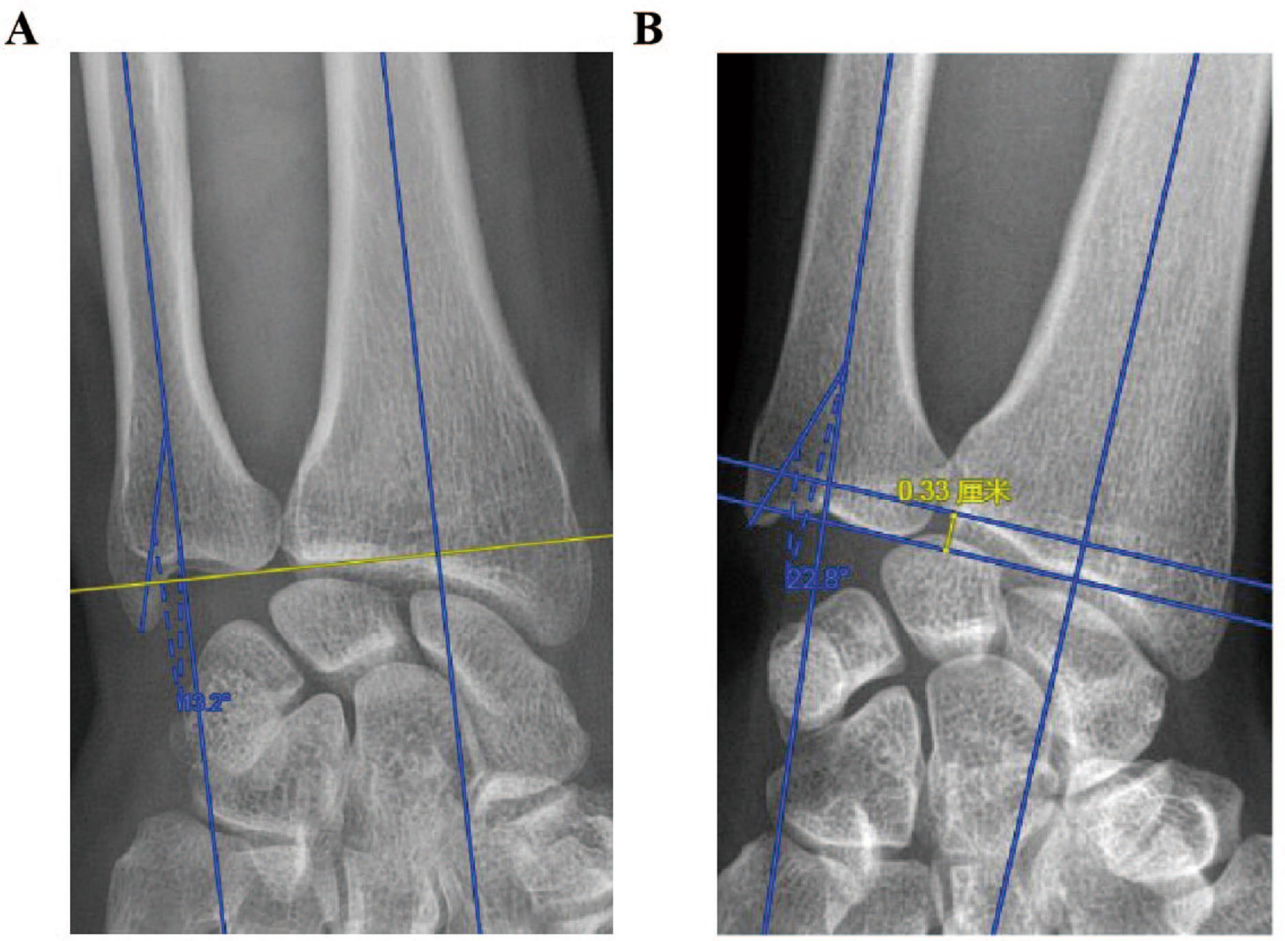
Radiographic measurement of the Ulnar Styloid Abduction Angle (USAA) on strictly controlled true posteroanterior (PA) wrist radiographs. **(A)** A representative radiograph from the control group (intact TFCC) demonstrates a neutral ulnar variance (UV = 0 mm) and a physiologically normal USAA of 13.2°. **(B)** A representative radiograph from the case group (Palmer IB tear) exhibiting a pathologically increased USAA of 22.8°, visually illustrating the severe radial abduction of the styloid process, which alters the coronal isometric constraints of the foveal attachment.

This study measured the ulnar styloid abduction angle (USAA). The angle was measured in the coronal plane between the established anatomical axis of the ulnar shaft, defined as the line connecting the midpoints of the cortical bone at 2 cm and 4 cm ulnar head articular surface, and the axis of the ulnar styloid, defined as the line connecting the midpoint of the styloid base to its tip. Angles deviating radially were recorded as positive values. Concurrently, the classic UV was measured using the method of perpendiculars, with positive variations recorded as positive values and negative variations as negative values. Specific standardised radiographic localisation and reference lines, as well as a visual comparison of normal morphology and high-risk abnormal deviation patterns, are detailed in Figure 1.

### 2.4 Clinical data collection

Baseline demographic and clinical characteristics of patients were extracted from electronic health records to serve as potential confounding factors in the multivariate analysis. The variables extracted included age, sex, body mass index (BMI), whether the affected side was the dominant hand, and the presence of comorbidities such as a history of smoking, diabetes, and hypertension.

### 2.5 Statistical analysis

All statistical analyses were performed using SPSS 26.0 software and the R programming environment. Missing values for covariates (BMI and smoking status) were handled using multiple imputation by chained equations (MICE) to generate five imputed datasets. Statistical analyses were performed on each imputed dataset separately. The resulting point estimates, standard errors, and odds ratios (ORs) were then pooled using Rubin’s rules to produce a single set of integrated results and associated *p* values, accounting for the uncertainty introduced by the imputation process. The distribution of continuous variables was assessed using the Shapiro–Wilk test; continuous variables with skewed distributions, such as age and BMI, were presented as the median and interquartile range (IQR), with intergroup comparisons performed using the Mann–Whitney U test. Categorical variables were presented as frequencies and percentages, and comparisons were made using the Pearson chi-square test or Fisher’s exact test. The reliability of the USAA measurement was assessed by calculating the inter-observer and intra-observer intraclass correlation coefficients (ICC) using a two-way mixed-effects model. A stratified multivariable binary logistic regression model was constructed to identify independent predictors for TFCC Palmer Grade IB tears. After adjusting for age, sex, BMI, involvement of the dominant hand, and common comorbidities, the adjusted odds ratios (aOR) and their 95% confidence intervals (CI) for USAA and UV were calculated. The diagnostic predictive performance of USAA was assessed using receiver operating characteristic (ROC) curves and their area under the curve (AUC), and the optimal clinical cut-off value was determined using the Youden index. All tests were two-sided, and *p* < 0.05 was considered statistically significant.

## 3 Results

### 3.1 Patient cohort, baseline characteristics, and propensity score matching

This study ultimately included 362 patients who met strict radiological and clinical quality control criteria. Of these, 110 cases constituted the case group with arthroscopically confirmed Palmer Grade IB TFCC tears, whilst 252 cases formed the control group with intact TFCC confirmed by high-resolution MRI and follow-up.

Given the limited missing data inherent in retrospective data collection (2.8% missing for BMI and 9.7% for smoking history), and after ruling out the possibility of complete random missing data using Little’s MCAR test (*p* = 0.031), this study employed the Multiple Interpolation by Chained Equations (MICE) method to generate five complete datasets for subsequent baseline and regression analyses.

In the entire cohort, the median age in the case group was lower than that in the control group (28 years [IQR, 23–35] vs. 31 years [IQR, 24–41], *p* = 0.042), and there was a tendency for a higher proportion of dominant-hand involvement and a higher smoking rate. To eliminate potential confounding bias introduced by baseline imbalances in age and other clinical characteristics between the two groups, this study performed 1:1 propensity score matching (with a cut-off value set at 0.05). Following matching, a pseudo-cohort comprising 190 patients (95 in each group) was successfully constructed. In the PSM cohort, the standardised mean difference (SMD) for all baseline covariates was less than 0.10, and clinical confounders between the two groups were well balanced statistically (Table 1).

**Table 1:** Comparison of baseline characteristics and anatomical parameters between the full cohort and the propensity score-matched (PSM) cohort.

Variable	Entire cohort (*n* = 362)		*p*-value	PSM matching queue (*n* = 190)		*p*-value
	Case group (*n* = 110)	Control group (*n* = 252)		Case group (*n* = 95)	Control group (*n* = 95)	
Clinical features						
Age (years), median [IQR]	28[23–35]	31[24–41]	0.042	29 [24–36]	29[25–37]	0.815
Gender (male), *n* (%)	60 (54.5%)	121 (48.0%)	0.258	51 (53.7%)	50(52.6%)	0.881
BMI (kg/m^2^), mean ± SD	24.5 ± 2.9	24.1 ± 3.1	0.252	24.4 ± 3.0	24.3 ± 2.8	0.822
Dominant hand affected, n (%)	66 (60.0%)	126 (50.0%)	0.081	55 (57.9%)	56(58.9%)	0.885
Smoking history, *n* (%)	33 (30.0%)	50 (20.0%)	0.054	28 (29.5%)	26(27.4%)	0.749
Anatomical parameters						
USAA (degrees), mean ± SD	21.0 ± 4.0	14.8 ± 3.8	<0.001	20.8 ± 3.9	15.1 ± 3.7	<0.001
UV (mm), mean ± SD	+1.5 ± 1.6	−0.1 ± 1.4	<0.001	+1.4 ± 1.5	+0.2 ± 1.3	<0.001

Continuous variables are expressed as mean ± standard deviation (SD) or median [interquartile range, IQR]. Categorical variables are expressed as frequency (percentage). *p*-values were pooled from five imputed datasets using Rubin’s rules to account for missing data uncertainty. USAA, ulnar styloid abduction angle; BMI, body mass index.

### 3.2 Comparison of radiographic parameters and measurement reliability

For the newly defined two-dimensional radiographic parameter USAA, the inter-observer intraclass correlation coefficient (ICC) was 0.92 (95% CI, 0.89–0.94), and the intra-observer ICC was 0.95 (95% CI, 0.93–0.97). The inter- and intra-observer ICCs for UV were 0.88 and 0.91, respectively. This indicates that, provided strict quality control for the ‘true anteroposterior’ view is maintained, USAA demonstrates excellent reproducibility on standard radiographs.

**Figure 2:**
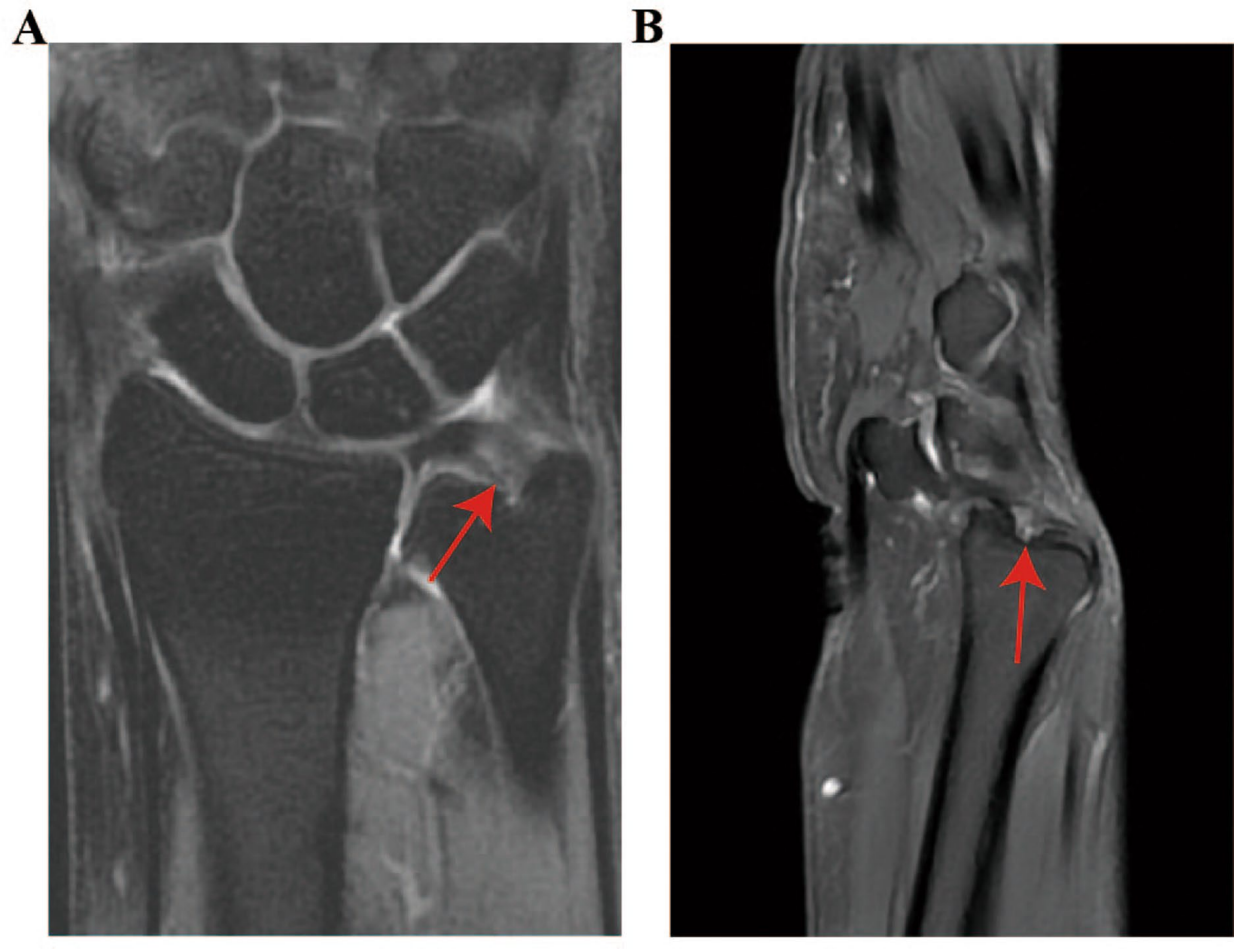
Representative high-resolution 3.0T MRI of a typical Palmer Type IB TFCC tear. **(A)** Coronal fat-suppressed T2-weighted image demonstrates a complete avulsion of the deep radioulnar ligament at the ulnar fovea. **(B)** Corresponding sagittal image provides an orthogonal perspective of the radiocarpal joint, further confirming local inflammatory exudation and loss of structural tension in the dorsal/palmar ligamentous complex.

In a direct comparison of anatomical morphology, the mean USAA in the case group was significantly greater than that in the control group in both the full cohort and the PSM-matched cohort (PSM cohort: 20.8° ± 3.9° vs. 15.1° ± 3.7°; *p* < 0.001). Concurrently, UV in the case group was significantly skewed towards positive values. To further correlate these radiographic findings with actual soft tissue pathology, representative high-resolution 3.0T MRI images were utilized to confirm the presence of Palmer Type IB tears in patients with pathologically increased USAA (Figure 2).

### 3.3 Independent predictor and diagnosis of multicollinearity

Prior to constructing the multivariate binary logistic regression model, a diagnosis of multicollinearity revealed that the variance inflation factors (VIF) for both USAA and UV were less than 1.5, with a tolerance greater than 0.6, confirming that the anatomical parameters included in the model did not present a significant risk of multicollinearity.

In the multivariate analysis after adjusting for confounding factors (Table 2), both USAA (aOR = 1.37; 95% CI, 1.25–1.51; *p* < 0.001) and ulnar deviation (aOR = 1.41; 95% CI, 1.13–1.76; *p* = 0.003) were both confirmed as independent bony factors associated with Palmer Type IB tears. Notably, in the multivariate model, age showed a marginally significant negative association (aOR = 0.97; *p* = 0.095), suggesting that younger individuals may face a higher risk of isolated deep ligament tears after controlling for anatomical abnormalities.

**Table 2:** Multivariate binary logistic regression analysis predicting TFCC Palmer Type IB tears.

Predictors	Unadjusted OR (95% CI)	*p*-value	Adjusted aOR (95% CI)	*p*-value
USAA (for each 1° increase)	1.48 (1.35–1.63)	<0.001	1.37 (1.25–1.51)	<0.001
UV (increase by 1 mm each)	1.62 (1.37–1.91)	<0.001	1.41 (1.13–1.76)	0.003
Age (years)	0.96 (0.93–0.99)	0.021	0.97 (0.93–1.01)	0.095
Gender (male vs. female)	1.30 (0.83–2.05)	0.258	1.15 (0.64–2.06)	0.632
BMI (kg/m^2^)	1.04 (0.96–1.13)	0.354	1.07 (0.96–1.19)	0.252

OR, odds ratio; aOR, adjusted odds ratio; CI, confidence interval. The multivariate model included USAA, UV, age, sex, BMI, and dominance of the affected hand as covariates; the model demonstrated good overall goodness of fit.

### 3.4 Non-linear dose–response relationship

**Figure 3:**
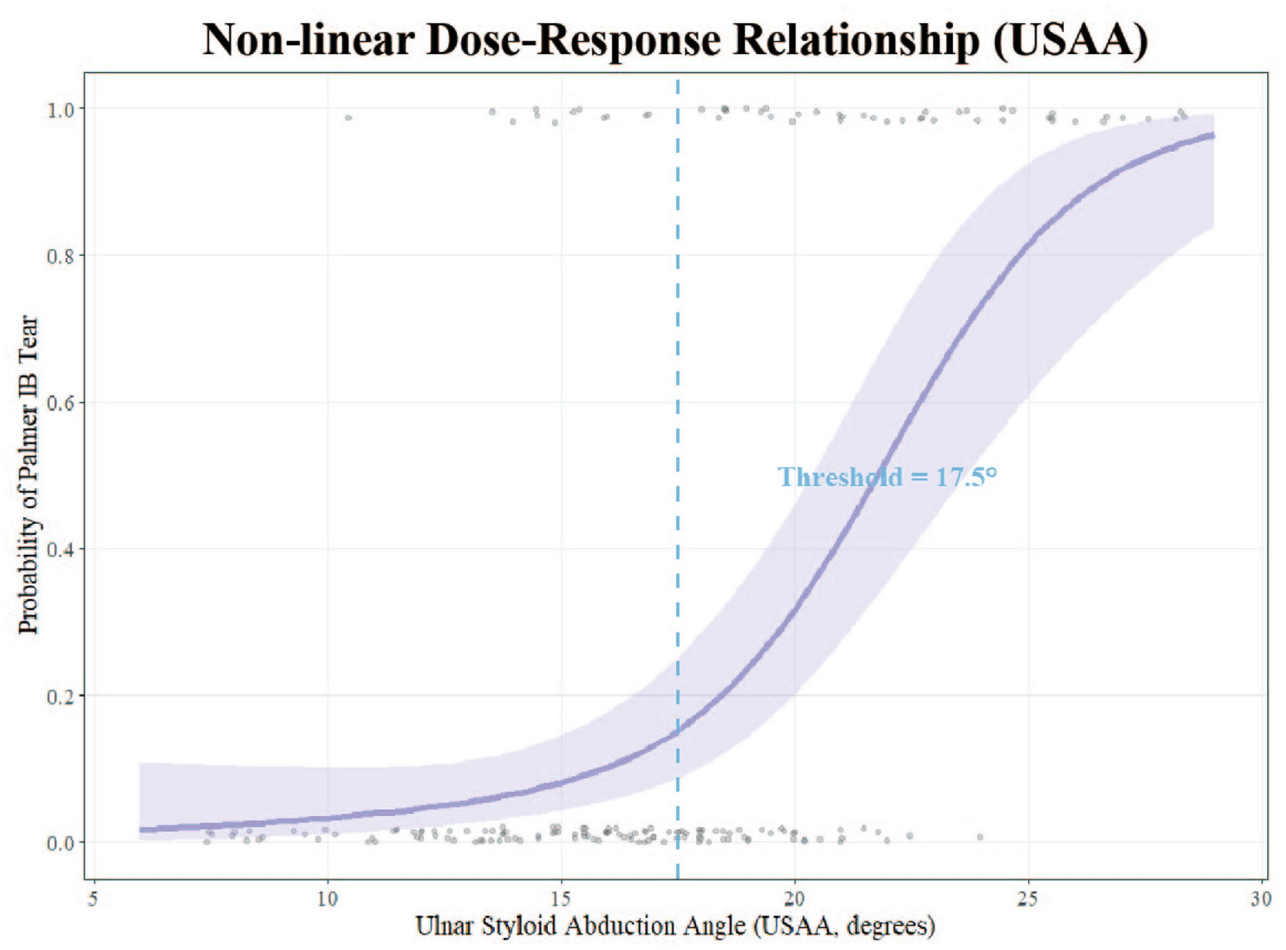
Non-linear dose–response relationship between USAA and the risk of Palmer Grade IB TFCC tears, as simulated using restricted cubic spline fitting. The scatter plot represents the distribution of actual cases, the solid purple line represents the fitted probability trend line, and the shaded area indicates the 95% confidence interval. When the USAA exceeds the critical threshold of 17.5° (blue dotted line), the probability of a tear exhibits a steep non-linear increase.

To avoid potential fitting biases associated with traditional linear models, this study employed nodal Restricted Cubic Spline (RCS) logistic regression to fit the curve and conducted an in-depth analysis of the dose–response relationship between the continuous variable USAA and the risk of tear. Analysis revealed a significant non-linear association between USAA and the risk of Palmer Grade IB tears. The risk curve was relatively flat when USAA was less than 16°, but once USAA exceeded an anatomical threshold of approximately 17.5°, the adjusted probability of deep fibre tears exhibited a steep, exponential rise (Figure 3).

### 3.5 Diagnostic predictive performance, internal validation, and model calibration

In the discriminatory assessment, the apparent area under the receiver operating characteristic curve (Apparent AUC) for univariate prediction of Palmer Grade IB tears using USAA was 0.857. To address the potential risk of model overfitting, internal validation was performed using the bootstrap method with 1,000 resamples. The optimism-corrected AUC was 0.849, indicating that this indicator retains robust diagnostic value in unseen data.

**Figure 4:**
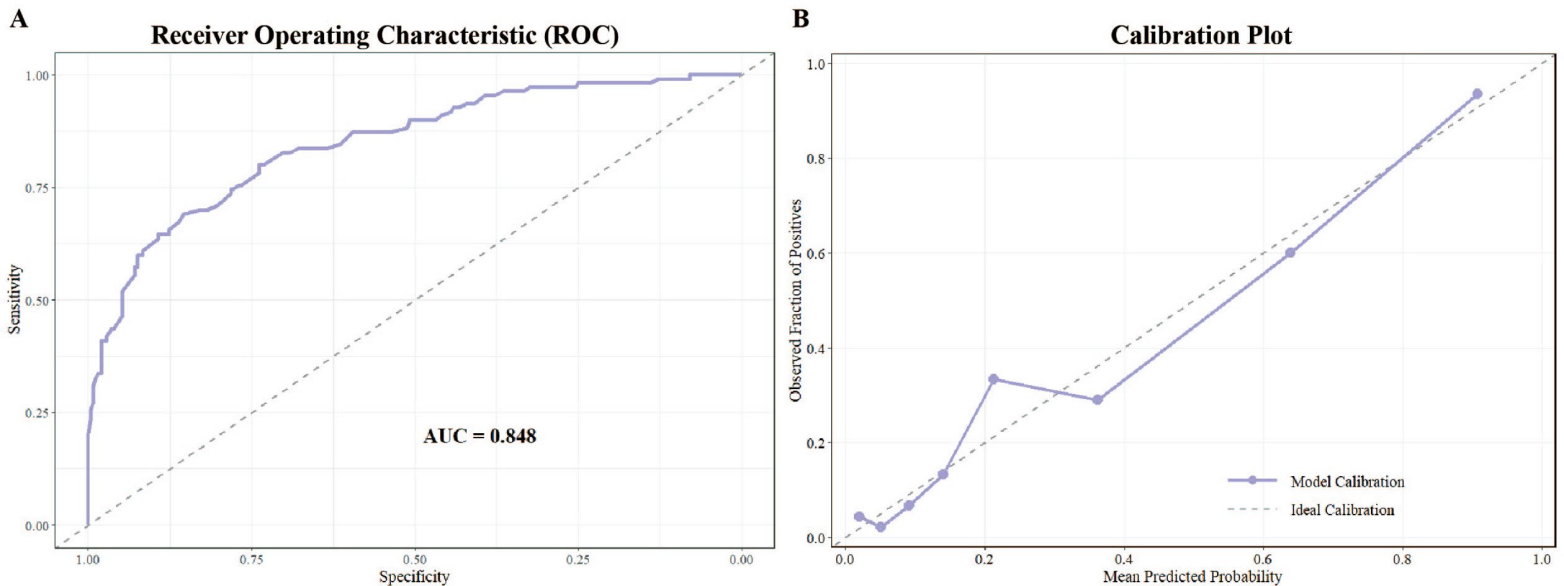
Diagnostic performance and model calibration plots for USAA. **(A)** Receiver operating characteristic (ROC) curve. The apparent AUC is 0.857, and the AUC after optimism correction is 0.849. **(B)** Calibration curve. The dotted diagonal line represents perfect calibration, whilst the solid line passing through the origin indicates the actual fit of the USAA model in this study. The close alignment of the prediction curve with the diagonal line confirms the accuracy of the model’s probabilistic predictions.

In accordance with the principle of maximising the Youden index, the optimal clinical cut-off value for USAA was established as 17.5° (sensitivity 81.8%, specificity 78.2%). Furthermore, graphical analysis of the calibration plot confirmed a very high degree of consistency between the model’s predicted probability of occurrence and the actual observed incidence rate (Figure 4).

### 3.6 Clinical net benefit and decision curve analysis

**Figure 5:**
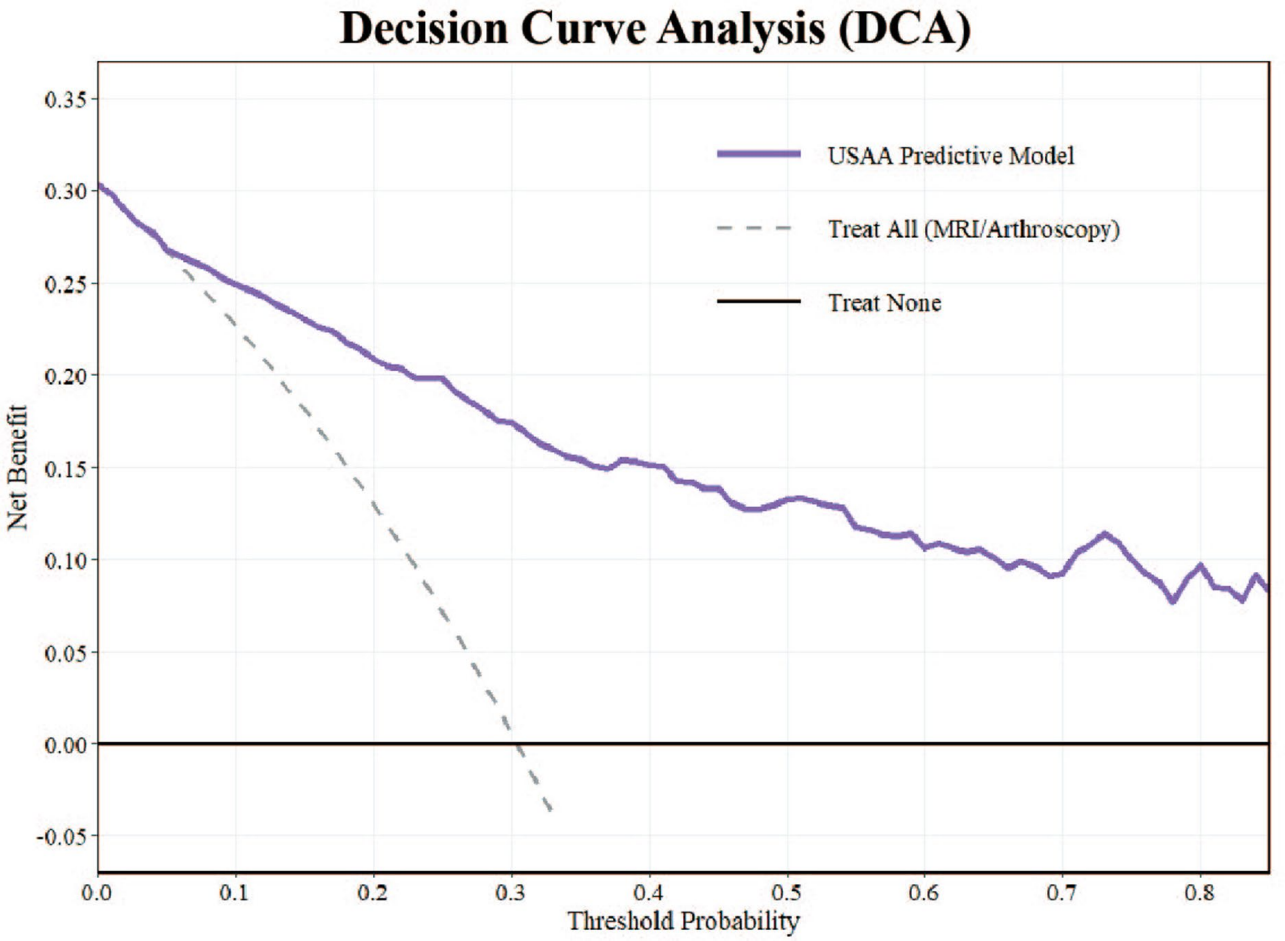
Decision curve analysis evaluating the clinical utility of the USAA model. The x-axis represents the threshold probability, and the y-axis represents the clinical net benefit. The solid purple line represents the net benefit of clinical decision-making based on the USAA indicator, which is significantly higher than that of the reference strategies “full intervention” (grey dashed line) and “no intervention” (black baseline), confirming its practical value in optimising early triage pathways in outpatient settings.

Given that high statistical predictive performance does not necessarily translate into clinical utility, this study further employed decision curve analysis (DCA) to quantitatively assess the clinical decision-making value of the USAA model. The DCA results indicate that, across an extremely wide range of threshold probabilities (approximately 10 to 85%), the clinical net benefit of using the USAA-based predictive model to guide initial intervention decisions (i.e., determining whether patients require further advanced investigations or interventions) consistently remains significantly higher than that of the extreme strategies of ‘treating all patients’ or ‘treating none’ (Figure 5).

## 4 Discussion

This study established the USAA as an independent and highly significant coronal morphological predictor of Palmer Type IB TFCC tears. By integrating PSM with RCS analysis in a rigorously controlled clinical cohort, we demonstrated the pathogenic relevance of this two-dimensional anatomical parameter independently of classic UV. Furthermore, we revealed a non-linear threshold effect between abnormal coronal plane deviation and the risk of deep ligament tears. Supported by the high clinical net benefit confirmed via DCA, USAA demonstrates significant potential as a first-line screening indicator for ulnar-sided wrist pain.

Traditional aetiological theories of ulnar-sided wrist pain rely heavily on changes in axial stress induced by longitudinal anatomical abnormalities. While UV has been extensively studied for its role in increasing radiocarpal load transmission and inducing ulnar impaction syndrome (9, 18), our multivariate model and PSM results clearly indicate that the pathomechanics of USAA differ fundamentally from those of UV. Rather than acting synergistically, they are mutually independent predictors with distinct pathomechanical implications (19).

If the pathological core of UV is axial bony impingement, we propose a potential pathomechanical hypothesis that the core of USAA might lie in the failure of isometric constraints in the coronal plane (20–22). To explore this plausible explanatory model, it is essential to consider the three-dimensional biomechanics of the DRUJ and the micro-mechanical properties of the ligament-bone junction. The deep radioulnar ligament is firmly attached to the ulnar fovea and serves as the core rotational pivot of the DRUJ. In a natural, neutral anatomical configuration, the normal base of the ulnar styloid provides a physiological mechanical fulcrum aligned with the stress lines of the deep fibres, ensuring that the ligament primarily bears uniform tensile stress under rotational loads (23). Theoretically, when the ulnar styloid process undergoes extreme radial abduction, that is, when the USAA is significantly increased, this local spatial configuration might be reshaped (13, 24). We speculate that the abnormally oblique bony prominence could potentially force a shift in the attachment trajectory of the deep fibres. Consequently, during extreme ulnar deviation or high-torque rotation, tensile loads that would normally be transmitted along the ligament’s long axis might be partially converted into non-physiological shear stresses and localised avulsion forces directed at the ligament’s foveal insertion (25, 26). Given that the uncalcified fibrocartilage layer exhibits extremely low tolerance to shear stress, such chronic abnormal shear could potentially disrupt the collagen network and impede matrix remodelling, which may lead to microscopic degeneration at the interface (27–31). The non-linear threshold revealed by the RCS curve in our study provides macroscopic clinical support for this hypothesized mechanism.

Our analysis indicates that the risk of tear does not increase linearly with USAA, but rather rises exponentially once a narrow critical range of 16.5°–17.5° is exceeded. This dose–response curve suggests that 17.5° likely represents a biomechanical tipping point. Once the coronal plane deviation exceeds this threshold, the compensatory capacity of the local soft tissues to withstand shear stress may be overwhelmed, predisposing the insertion site to structural failure. Furthermore, our multivariate model identified a marginally significant trend (*p* = 0.095), suggesting a possible association between younger age and an increased likelihood of Palmer Type IB tears (aOR = 0.97). While this finding does not reach strict statistical significance and should be interpreted with caution, it provides a preliminary basis to consider that isolated deep TFCC tears may not be driven solely by age-related degeneration. We hypothesize that this observed trend could be related to the higher frequency of high-torque wrist movements, such as those in racket sports, typically performed by younger individuals. In such high-demand scenarios, an inherent coronal plane susceptibility (high USAA) might interact with intensive acquired shear loads, potentially contributing to the premature failure of the ligamentous insertion (13, 32). However, these observations remain speculative and require validation in larger, prospectively designed cohorts.

Regarding translation into clinical practice, the DCA results carry substantial clinical relevance. Although high-resolution 3.0T MRI or 3D-CT provides superior spatial localization, standard 2D wrist radiographs remain the primary imaging modality in most initial orthopaedic consultations and emergency settings. The DCA objectively demonstrates that utilizing the simple radiographic cut-off of USAA ≥ 17.5° for initial triage offers substantial net benefits across a wide range of threshold probabilities. This parameter can empower primary care physicians to rapidly identify high-risk indicators of deep ligament instability in patients presenting with non-specific ulnar-sided wrist pain. Consequently, this enables more targeted referrals for advanced imaging or early arthroscopic intervention, which may effectively prevent the irreversible DRUJ osteoarthritis associated with delayed diagnosis.

This study has several limitations. First, due to its retrospective design, although advanced statistical methods like MICE and PSM maximized the balance of known confounders, unobserved variables—such as patients’ long-term specific exercise frequency—could not be quantified. Second, we must explicitly acknowledge the inherent limitations of our control group selection criteria. Although the use of high-resolution 3.0T MRI combined with long-term clinical follow-up is a pragmatically reasonable approach, it remains secondary to the diagnostic “gold standard” of arthroscopy. Consequently, our control cohort may have included patients with occult or functionally incompetent deep foveal TFCC lesions that were radiologically undetectable. Such potential for misclassification bias could have influenced the statistical discrimination of the USAA threshold. Third, as a 2D parameter, USAA carries an inherent risk of projection distortion. Although we established exceptionally stringent criteria for “True PA” images, this rigor led to the exclusion of non-compliant radiographs, potentially introducing selection bias. Future research should explore deep learning algorithms for 2D-to-3D parameter conversion mapping. Finally, the “tensile-shear stress conversion” mechanism proposed herein remains a theoretical deduction based on clinical epidemiological thresholds. Future *in vivo* studies directly measuring the strain map at the ulnar fovea across a gradient of USAA values—either through individualized 3D finite element analysis or kinematic tension testing on cadaveric specimens—are required to validate this hypothesis.

## 5 Conclusion

In summary, an increased ulnar styloid abduction angle (USAA) is not only an independent bony predictor for Palmer Type IB TFCC tears, but also exhibits a non-linear biomechanical threshold at 17.5° that triggers an increased risk of pathology. This anatomical abnormality in the coronal plane is independent of classic longitudinal UV, and its underlying mechanism may lie in the induction of micro-shear mechanical failure at the ligament-bone junction. Given its extremely high measurement reliability and net clinical decision-making benefit, the USAA is expected to become a standard screening radiographic parameter for optimising first-line diagnostic triage in patients with ulnar wrist pain.

## Data Availability

The original contributions presented in the study are included in the article/supplementary material, further inquiries can be directed to the corresponding authors.

## Glossary

aOR: Adjusted odds ratio
AUC: Area under the receiver operating characteristic curve
BMI: Body mass index
CI: Confidence interval
DCA: Decision curve analysis
DRUJ: Distal radioulnar joint
ECU: Extensor carpi ulnaris
ICC: Intraclass correlation coefficient
IQR: Interquartile range
MICE: Multiple imputation by chained equations
MRI: Magnetic resonance imaging
OR: Odds ratio
PA: Posteroanterior
PACS: picture archiving and communication system
PSM: Propensity score matching
RCS: Restricted cubic spline
ROC: Receiver operating characteristic
SD: Standard deviation
SMD: Standardised mean difference
TFCC: Triangular fibrocartilage complex
USAA: Ulnar styloid abduction angle
UV: Ulnar variance
VIF: Variance inflation factor
